# The prospective acceptability of preventative IV bisphosphonate therapy prior to fracture: Perspectives of young people with Duchenne muscular dystrophy, parents and health professionals

**DOI:** 10.1371/journal.pone.0324124

**Published:** 2025-06-02

**Authors:** Danielle C. Mountain, Nicola Crabtree, Claire L. Wood, Sze Choong Wong, Lucy Bray

**Affiliations:** 1 Faculty of Health, Social Care and Medicine, Edge Hill University, Ormskirk, United Kingdom; 2 Department of Diabetes and Endocrinology, Birmingham Women’s and Children’s NHS Trust, Birmingham, United Kingdom; 3 Newcastle University Translational and Clinical Research Institute, Newcastle upon Tyne, United Kingdom; 4 Bone, Endocrine, Nutrition Research Group in Glasgow, Human Nutrition, University of Glasgow, Glasgow, United Kingdom; 5 Department of Paediatric Endocrinology, Royal Hospital for Children, Glasgow, United Kingdom; Aga Khan University, PAKISTAN

## Abstract

Young people with Duchenne muscular dystrophy (DMD) commonly experience osteoporosis and fractures which can lead to chronic pain and reduced quality of life. Initiating IV bisphosphonate therapy prior to first fracture may be a logical primary preventative approach given the extent and related morbidity of osteoporosis, although there is limited evidence for this. This qualitative study using semi-structured interviews and focus groups, aimed to explore the opinions and prospective acceptability of young people with DMD, parents and health professionals in the UK on initiating bisphosphonate therapy prior to first fracture. Four boys with DMD (aged 15–17 years) and 20 parents participated in semi-structured interviews. Twenty-seven health professionals involved in the care of young people with DMD participated in focus groups. A framework analysis was conducted. Three categories were identified which represented a continuum of opinions on the endorsement of preventative bisphosphonate therapy: 1) “It buys them time”, endorsement of preventative bisphosphonate therapy; 2) Uncertainty and the importance of “choice”; and 3) “Worry about... starting bisphosphonates even earlier”, not endorsing the use of preventative bisphosphonate therapy. Young people with DMD and parents discussed a range of opinions about the prospective acceptability of IV bisphosphonate as preventative therapy, highlighting the importance of family choice before initiation of therapy. Health professionals called for future research exploring the risks and benefits of preventative IV bisphosphonate therapy for young people with DMD to inform clinical practice.

## Introduction

Duchenne muscular dystrophy (DMD) is a rare, life-limiting condition which is characterised by progressive loss of muscle and weakness. Corticosteroids are the standard of care for children/young people with DMD as a disease modifier to slow the progression of the disease [1,2]. Long-term corticosteroid use and progressive myopathy contributes to a significant risk of osteoporosis leading to low trauma fractures of the long bone and vertebral fractures [3–7]. Symptomatic fractures (long bone and vertebral fractures presenting with significant back pain) have been reported to be approximately four times higher in DMD compared with healthy controls [8]. Vertebral fractures can occur as early as 6 months from corticosteroid initiation [9]. Approximately 75% of children/young people who take daily corticosteroid for a duration of 8.5 years will develop long bone or vertebral fractures [10]. Long bone fractures often lead to premature loss of ambulation [3] and may rarely be complicated by fat embolism syndrome which has been associated with fatalities [11]. Severe and progressive vertebral fractures may lead to chronic back pain which impairs quality of life [9].

The current International Care Considerations for DMD (2018) recommend the initiation of intravenous (IV) bisphosphonate treatment following identification of vertebral fractures from lateral spine imaging or low trauma long bone fractures [12]. Bisphosphonates are stable analogs of inorganic pyrophosphate with a strong affinity for hydroxyapatite in the bone matrix, particularly in areas of active remodeling. Bisphosphonates act on reducing bone resorption by inhibition of the mevalonic acid pathway, disrupting key post-translational modifications required for osteoclast survival and function, leading to osteoclast apoptosis [13].

This secondary preventative approach in DMD leads to improvement in bone density and stabilisation of the severity of vertebral fractures [14–17]. However, there are side-effects of IV bisphosphonates which include the relatively common and often severe first infusion reaction [18]. This reaction can be worsened by precipitating adrenal crisis in those on corticosteroid and rare complications like rhabdomyolysis and intracardiac thrombosis [19–21].

Whilst not recommended in the current standards of care, IV bisphosphonate therapy *prior* to first fracture has been discussed and debated as a potentially valuable preventative approach [22]. The rationale for moving the dial to a primary prevention approach may seem logical given that fractures occur in many of these young people, especially those treated with daily corticosteroid. In addition, progression to moderate or severe vertebral fracture can occur despite annual routine lateral spine imaging, even if prior imaging results were normal or showed minimal height loss [22]. There is increasing literature on the use of bisphosphonates in DMD [17]. However, to date, there are limited well powered studies that have included a fracture naive population. The impact of IV bisphosphonates on fracture prevention when used as a primary preventative agent prior to first fracture is still unknown. In developing evidence-based healthcare, it is important to examine acceptability [23] to help guide future research to examine efficacy and effectiveness of therapy. Therefore, it is crucial to consider the perspectives of children/young people with DMD, their parents and health professionals on the prospective acceptability [23] of preventative IV bisphosphonate therapy.

This study aimed to explore the opinions of children/young people with DMD, their parents (including carers) and health professionals on the prospective acceptability of initiating IV bisphosphonate therapy prior to first fracture.

## Materials and methods

### Public involvement

Public involvement aimed to inform the choice of methods and recruitment strategies. The research team consulted with two parents of children/young people with DMD who were identified through a UK patient organisation (Duchenne UK). The public involvement is reported according to the GRIPP-2 (Guidance for Reporting Involvement of Patients and the Public) short form [24]. Consultation occurred over video call on two occasions. Parents identified the need for interviews with parents and flexible data collection methods to enable children/young people to take part (including the choice to use activity sheets during interviews). They also helped to develop sensitive wording around vertebral fractures particularly for those children/young people who had not yet experienced a fracture. The parents guided the development of the interview questions and the recruitment flyer to ensure it was visually appealing and the options for involvement were highlighted.

### Design

A qualitative design was implemented to examine participants’ perspectives of bone health monitoring and treatment in DMD. This paper focuses specifically on their opinions towards initiating IV bisphosphonate therapy prior to first fracture. Researchers conducted recruitment for semi-structured interviews (in person or online) with young people with DMD and/or parents between 5^th^ September 2023–26^th^ March 2024. Remote online focus groups were undertaken with health professionals who cared for children/young people with DMD (recruitment between 28^th^ November 2023–21^st^ February 2024) to facilitate discussion and exploration of views between health professionals. The Consolidated Criteria for Reporting Qualitative Research (COREQ) was completed for reporting purposes (S1 Table) [25]. Ethics approval was granted by the corresponding author’s University Research Ethics Committee (ETH2223–0325).

### Participants

#### Children, young people and parents.

Children/young people with DMD were eligible to take part if they were aged 7–18 years old and lived in the UK. Previous vertebral and/or long bone fracture was not a requirement. Parents (including carers) of children/young people with DMD were also invited to share their views. Children/young people and their parents could take part independently from each other. Participants had to be able to understand and communicate in English. Children/young people had to be able to reflect on their experiences either independently or assisted by their parent.

The research team distributed study flyers on their professional social media accounts and contacted gatekeepers at relevant charities (Duchenne UK, Action Duchenne, Muscular Dystrophy UK), parent networks and support groups. Gatekeepers distributed recruitment flyers internally within their networks. Parents contacted the research team to express an interest in the study. Convenience sampling was followed by purposeful sampling to achieve maximum variation by targeting particular geographical areas and service provision. Snowball sampling was encouraged by asking parents to share details about the study with other parents of children/young people with DMD they thought might be interested in taking part.

#### Health professionals.

Health professionals were eligible to participate if they cared for children/young people with DMD in the UK, including but not limited to: neuromuscular clinicians, bone/endocrine specialists, nurses, physiotherapists, dietitians and psychologists.

Recruitment flyers were distributed via Duchenne UK’s circulation list of clinicians through the DMD Care UK project (https://dmdcareuk.org/). Clinicians within this list had agreed to be contacted for updates relating to the DMD standards of care and surveys pertaining to the care of children/young people with DMD. Recruitment flyers were also distributed via the research team’s professional social media accounts.

### Data collection

The research team developed three flexible interview schedules to explore the views of children/young people, parents and health professionals separately. Open-ended questions explored participants’ perspectives on the prospective acceptability and use of bisphosphonate treatments prior to first fracture. Two female researchers (DCM, MSc, a postdoctoral research assistant, and LB, PhD, a Professor in Child Health Literacy) who were experienced in health research and qualitative research methods, facilitated and conducted the interviews and focus groups. The researchers explained the study aims and rationale to participants during recruitment and in the consent process. Young people (aged 16 and above), parents and health professionals signed an informed consent form prior to the interview or focus group beginning. Young people (aged 15 or below) signed an informed assent form and their parent provided additional written informed consent. Data collection continued until data sufficiency was achieved, for example breadth and depth of the data [26,27].

#### Interviews.

Interviews were undertaken online (via Teams) or in person at participant’s homes if they lived local to the research team. Prior to interviews, the research team sensitively checked with parents how much knowledge their child/young person had about vertebral or long bone fractures. They also ascertained whether any adjustments were needed to help their child/young person join in the interview (e.g., provision of activity sheets during the interview). Interview questions were adapted to discuss preventative measures for bone health more generally for those young people who had not experienced or had no knowledge of vertebral or long bone fractures. The researchers provided participants with example interview questions prior to the interview. Interviews were coordinated so that parents could speak at a time without their child present. Young people chose whether they wanted their parent present during their interview. Coordinating interviews separately aimed to ensure that both young people and parents could openly share their views and issues of importance to them [28,29]. Interviews were audio-recorded, transcribed verbatim and lasted between 12–20 minutes with boys and 20–45 minutes with parents. A £15 shopping voucher was provided to the boys to recognise their contributions.

#### Focus groups.

Four focus groups were held online (via Teams) with between four to eight health professionals per group. Online synchronous focus groups were chosen as professionals are comfortable and familiar sharing their experiences and ideas in an online environment [30] and the shared experience of caring for children/young people with DMD, whilst holding different views of bone health management prompted and stimulated the discussion. Multiple focus groups were held to protect against a dominant voice shaping the data collected [30] and the discussion was moderated to ensure every participant was provided with sufficient opportunity to express their opinion. Each focus group was planned to include a mix of multi-disciplinary professionals, to ensure a range of professional perspectives informed the discussion. The researchers (DCM, LB) co-moderated the group discussion, with one researcher leading the discussion and the second attending to comments in the chat function and making notes of contextual information about each clinical centre (the number of boys with DMD managed at each clinical centre and usual corticosteroid regimen prescribed). Focus groups were audio-recorded, transcribed verbatim and lasted between 66–83 minutes.

### Data analysis

Interviews and focus groups were analysed using framework analysis [31]. Two coders (DCM, LB) re-listened to audio-recordings, re-read transcripts and conducted independent preliminary open coding on the transcripts for the first five interviews and one focus group. This open coding was conducted using paper, pens and a Microsoft application (Word). The inductively derived codes were discussed, refined and agreed and grouped into categories and sub-categories into a working analytical framework [32] which reflected a continuum of opinions on preventative bisphosphonate therapy. This coding framework was then used to index the remaining dataset by one coder (DM), with refinements and additions being discussed throughout the coding process. The researchers charted the categories and participant quotes into a framework matrix held on a Microsoft application (Excel) and examined the framework for commonalities and divergences within and across datasets. Discrepancies or uncertainties which arose during analysis were acknowledged, debated and consensus reached. Sub-group analysis was not conducted to examine differences of perspectives between young people who had experienced or not experienced a long-bone fracture and their parents as there was wide variability in the characteristics and experiences in each group [33] and across the dataset as whole in respects to age, age of fracture, steroid medication, bisphosphonate therapy and ambulation.

## Results

Fifty-one participants took part in the study. Twenty-seven health professionals, twenty parents (17 mothers, 3 fathers) of 19 boys with DMD (median age = 12.0 years, range 7–17 years) and four boys (median age = 15.5 years, range 15–17 years) participated in interviews. Most boys were currently taking corticosteroids (n = 18, 95%), with the majority prescribed daily deflazacort (n = 14, 78%). Most used a wheelchair to some degree (n = 16, 84%). Fourteen (74%) boys had experienced a vertebral and/or long bone fracture, of which 13 (93%) were having IV bisphosphonate treatment. Table 1 contains demographic and clinical information about children/young people and parents.

**Table 1:** Demographic and clinical characteristics of young people and parents.

Participant number	Parent	Child’s or young person’s participation	Child’s or young person’s age at time of interview	Approx. age at diagnosis	Current corticosteroid regimen	Approx. age started corticosteroids	Wheelchair use	Experience of fracture? If yes, age and type
1	Father	–	13	3	Daily deflazacort	Not stated	Most of the time	8, vertebral
2	Mother	–	10	1	Daily deflazacort	8	All the time	8, vertebral
3	Mother and Father	–	9	5	Daily deflazacort	6-7	Most of the time	7, vertebral
4	Mother	Interview	15	8	10/10 deflazacort	9	All the time	No
5	Father	–	7	2	Daily vamorolone	5	Ambulatory	No
6	Mother	–	7	2	Daily deflazacort	4	Ambulatory	No
7	Mother	Interview	16	3	Daily deflazacort	5	Most of the time	14, vertebral
8	Mother	–	10	3	Daily deflazacort	4	Rarely	No
9	Mother	–	12	Not stated	Daily deflazacort	5	All the time	10, long bone
10	Mother	–	12	2	Daily deflazacort	4	Sometimes	10, vertebral
11	Mother	–	15	4	Daily deflazacort	6	All the time	8, long bone
12	Mother	–	14	2	Daily deflazacort	5	Most of the time	12, vertebral
13	Mother	–	14	5	Daily deflazacort	6	Most of the time	12, vertebral
14	Mother	–	11	5	Daily deflazacort	5	Sometimes	Uncertain of age, vertebral
15	Mother	–	14	3	–	5	All the time	11, long bone
16	Mother	–	9	7	10/10 prednisolone	7	Sometimes	8, vertebral
17	Mother	Interview	17	4	Daily prednisolone	5	All the time	12, vertebral
18	Mother	–	11	1	Daily deflazacort	8	Rarely	9, vertebral
19	Mother	Interview	15	6 months	Daily deflazacort	4	Ambulatory	No

Twenty-seven health professionals took part in focus groups; they included consultant paediatric endocrinologists/bone metabolism (n = 12), physiotherapists (n = 3), neuromuscular clinicians (n = 6, nurse specialists (genetics, endocrine/bone and clinical; n = 3), community paediatrician (n = 1), general practitioner (n = 1) and a clinical scientist (n = 1). The health professionals reported that their centres managed cohorts of between 6–260 boys with DMD. Within these centres, boys were on a mix of corticosteroids regimens (daily or 10 days on 10 days off; prednisolone or deflazacort), or no corticosteroids.

### Opinions on the endorsement of preventative IV bisphosphonate therapy

Three categories were constructed which reflected a continuum of opinions on the use of preventative IV bisphosphonate therapy. At one end of the continuum were those who endorsed the use of preventative IV bisphosphonate therapy as it “buys them time”. Towards the middle were those who saw the merit of preventative IV bisphosphonate therapy but advised it depends on “choice” and questioned the lack of robust evidence. At the other end were those who would not currently endorse the use of preventative IV bisphosphonate therapy. This was due to the burden associated with treatment and opinions that bone health protection and fracture prevention should go “beyond bisphosphonate”.

**Fig 1:**
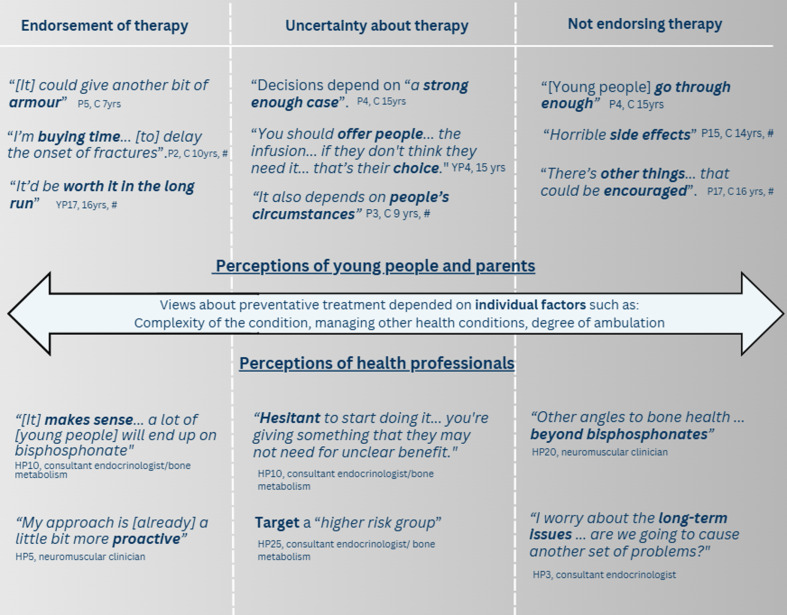
Participant’s opinions on the endorsement of preventative bisphosphonate therapy. Note. HP, health professional.

Fig 1 presents an illustration of the continuum of opinions towards the use of IV bisphosphonate as a preventative treatment. Categories are illustrated and supported by participant quotes (YP, young person; P, parent; HP, health professional, age of child and age of first fracture (#) are also noted)

#### Category one: “It buys them time”, endorsement of preventative IV bisphosphonate therapy.

##### 1.1 It “makes sense” to “armour” against fractures

Young people, parents and health professionals all discussed how preventative IV bisphosphonates should be used if they could delay or prevent fractures: *“I’d say it’s a good idea to do it before you have [a fracture], so then it can help prevent it from ever happening”* (YP17, aged 17, # aged 12).

Preventative treatment was discussed as of value as it *“could give another bit of armour, bit [of] protection*” (P5, C aged 7) to their child/young person and prevent or delay the subsequent consequences of experiencing fractures, particularly pain and limited mobility: *“I really wish we had [the preventative infusions]... I think he would still be walking quite well now if we had of done that... he wasn’t exercising as much because of the back pain”* (P12, C aged 14, #aged 12).

Parents questioned current clinical approaches for monitoring and managing bone health and fractures, which required a child/young person to experience a fracture before action was taken (i.e., bisphosphonate therapy was provided): “*how so incredulous that we would wait until he broke his, something, to then begin the treatment that would prevent him doing that... that’s mad surely there’s something we can do to prevent it”* (P8, C aged 10).

Both parents and health professionals discussed how preventative treatment would “*prevent stuff rather than firefighting”* (P9, C aged 12, # aged 10). A more proactive approach to initiation of treatment when there are signs of deterioration of bone health could help prevent the vertebral fracture cascade: *“I’m perhaps a little bit more proactive for example with children on daily dose or who are for example on deflazacort... if the bone density starts to go down, if there are some changes in the bone in your knowledge of this type of steroid that they are having... and the regime, maybe my approach is a little bit more proactive”* (HP5, neuromuscular clinician).

Parents and health professionals advised that preventative bisphosphonate therapy “*makes sense... because a lot of these will end up on bisphosphonate”* (HP10, consultant endocrinologist/bone metabolism). Parents compared preventative bisphosphonate therapy with other preventative medications that children/young people took for their health, such as the use of treatment to prevent DMD related cardiac dysfunction: *“for argument’s sake, for cardiology, we, I argued for prophylactic medication... he hasn’t got any issues with his heart at this point. So, I tend to think it did make a difference”* (P17, C aged 17, # aged 12).

##### 1.2 Focusing on the “end goal”

Parents and young people discussed how the burden and impact of the administration of preventative bisphosphonate therapy and “*feeling a bit rotten for a few hours*” (P14, C aged 11, #) could be balanced against the potential benefits of treatment: “*there is an end goal that we are trying to reach, or you know, a step that we are taking”* (P6, C aged 7).

Young people identified that the burden and impact of bisphosphonate therapy was short-lived compared to the impact fractures had on their life: *“it’d be worth it in the long run [having preventative IV bisphosphonate treatment] ‘cos then if you think about it, just a day every four months [for treatment] instead of it being four weeks out of everything and not being involved in school and stuff [for fractures]... I’d say it would be an improvement to that”* (YP7, aged 16, # aged 14).

Health professionals and parents discussed how the short-term burden of treatment could be reduced by having *“the first ones [infusions] are done in the central areas and the rest could be done more locally”* (HP5, neuromuscular clinician) or by having infusions *“coordinated… for like a whole day”* (P18, C aged 11, # aged 9) alongside other appointments.

The burden of hospital visits was “*no bother*” (P19, C aged 15) to some parents who considered “*travel and time... low down on the list*” (P9, C aged 12, # aged 10) compared to the benefits of the end goal in protecting their child’s/young person’s bone health. Parents had accepted that monitoring and managing their child’s/young person’s DMD and health required regular hospital visits or taking time off school: *“the burden of hospital appointments… it becomes part of your normal really. So we’re used to... driving an hour and a half to go to a 15-minute appointment… [it] becomes a normal way of life”* (P2, C aged 10, # aged 8).

#### Category two: Uncertainty and the importance of “choice” for preventative bisphosphonate therapy.

##### 2.1 “Strong enough case”, endorsement depends on the evidence and advice

Parents explained that any decisions they were involved in about preventative bisphosphonate therapy would depend on their access to and understanding of information about treatment. Specifically, decisions would depend on if the evidence presented a *“strong enough case”* as to *“why it’s recommended”* (P4, C aged 15) and whether health professionals could “*answer all the questions that we have, then we are in a position to make a decision”* (P6, C aged 7).

Parents discussed that they would be more likely to support preventative IV bisphosphonate use if health professionals or other families of young people with DMD recommended it: *“if this is what your professional clinicians are suggesting that you do and also if other people in the community are doing it as well. I think people would be happy to kind of go along with that”* (P2, C aged 10, # aged 8).

An important part for parents of participating in treatment decisions was that information was clearly explained and communicated to them in *“plain English*” (P1): *“if an endocrinologist says we want to start this infusion, where this weird name, which is an acid and we’re like what does that mean? What does it do? Why are we doing that? How does that fit in with everything else... this is what the advantages are. This is the disadvantages. This is what you know your first infusion there may be a reaction, but after that it tends to calm down”* (P1, C aged 13, # aged 8).

Health professionals discussed that without a strong evidence base, they were “*a bit hesitant to start doing it because you’re giving something that they may not need for unclear benefit.”* (HP10, consultant endocrinologists/bone metabolism). This led health professionals to highlight the need for future research exploring the use and effectiveness of preventative IV bisphosphonate therapy: *“sounds like an excellent idea [getting data on preventative bisphosphonate therapy]... whether it’s done as research or changing standard of care, some sort of ongoing monitoring or... registry or something prospective to monitor outcomes... would be important”* (HP5, neuromuscular clinician).

The benefit of preventative treatment needed to be strong, as health professionals raised the resource implications of implementation: *“we have more than 100 patients, how feasible is that? I treat more than 100 patients every six months with an IV bisphosphonate? Even if you tell me that is what needs to be done, actually it will take years to implement [in the] NHS to be able to support this”* (HP23, neuromuscular clinician).

Whilst much of the discussion focused on the administration of intravenous preventative treatment with bisphosphonates, both parents and health professionals discussed how if oral bisphosphonates *“was felt to be beneficial to them, I expect they [families] probably would be quite accepting of that”* (HP8, consultant endocrinologists/bone metabolism). Families would be likely to have “*a reasonable amount of interest”* (HP21, consultant endocrinologist) in oral bisphosphonates compared to intravenous bisphosphonates given as preventative treatment in DMD.

However, health professionals expressed concerns that *“there are problems potentially if [oral bisphosphonates are] not taken... quite strictly”* (HP8, consultant endocrinologists/bone metabolism) and required careful monitoring due to risks to the patient: *“I was always told you’ve got to be very, very careful if it’s not taken as prescribed, it can cause quite a few problems... it would worry me of the prophylactic use of the oral bisphosphonates… I’m not saying it hasn’t got a place, but... it would need to be very carefully monitored”* (HP9, endocrine bone nurse specialist).

The use of oral bisphosphonate therapy in improving bone outcomes (bone density and fracture) in DMD was also lacking in evidence: “*I don’t believe there’s very good evidence that oral bisphosphonates are as effective as IV in children... it would essentially require some research to know whether actually oral bisphosphonates do reasonably improve your bone density and prevent fracture”* (HP25, consultant endocrinologist/bone metabolism).

##### 2.2 Endorsement “depends on people’s circumstances”

Young people and parents discussed that families should be given a choice about preventative bisphosphonate treatment: *“I think you should offer people, like whether they’re walking or not, the infusion and if they don’t think they need it or want it, then that’s their choice in the end”* (YP4, aged 15). Parents described that these discussions and decisions should involve children/young people with DMD, and the outcome would depend on each individual. For some children/young people, parents explained “*if you explained it to him... he’d be quite happy [to have the therapy]*” (P14, C aged 11, #) whilst for others who struggled with invasive procedures, “*to even get him in there, I think would entail quite a lot of bribery and things*” (P8, C aged 10).

Some young people, parents and health professionals reported that a preventative approach might be less beneficial or not necessary for children/young people who were non-ambulatory or on certain corticosteroid regimens: *“for people that aren’t walking, I don’t really see the point... unless I was gonna be stupid and dangle my legs in front of my wheels. I’ve got no way of really breaking my bones unless I tip my chair up somehow”* (YP4, aged 15).

Parents discussed that attending for intravenous preventative bisphosphonate therapy may be dependent on individual family circumstances, such as work flexibility: “*it also depends on people’s circumstances... [my partner] works from home and I’m self-employed so we can get to the hospital for these things. I know if you’ve got two parents that are working, it is finding that time”* (P3, C aged 9, # aged 7). Health professionals’ opinions about the importance of individual circumstances in making decisions about preventative treatment were linked to a young person’s clinical presentation, such as targeting treatment to a “*higher risk group*” (HP25, consultant endocrinologists/bone metabolism) linked to a specified “*threshold*” (HP24, consultant endocrinologist): *“some have all these other issues that we’ve talked about, you know, the monitoring, needle phobia, just depressed and withdrawn and isolated and all those other things”* (HP1, paediatrician with interest in endocrinology).

#### Category three: “Worry about... starting bisphosphonates even earlier”, not endorsing the use of preventative bisphosphonate therapy.

##### 3.1 They “go through enough as it is”, the burden on children, young people and parents

Parents discussed how children/young people “*go through enough as it is”* (P4, C aged 15) without having additional preventative bisphosphonate therapies. It was important to parents that children/young people “*have fun, they don’t need to spend you know so much time in hospital”* (P18, C aged 11,# aged 9).

The short-term “*nasty”* (HP1, paediatrician with interest in endocrinology) and *“horrible side effects”* (P15) and potential long-term effects on the child’s/young person’s health was a concern for some parents and health professionals: “*I worry about the long-term issues if we’re going to start bisphosphonates even earlier. What’s that going to do sort of further down the line, are we going to cause another set of problems?”* (HP20, neuromuscular clinician).

Travelling to and attending hospital for preventative bisphosphonate therapy was described mostly by health professionals as being “*quite onerous*” (HP21, consultant endocrinologist) for families. Some young people and parents identified the time travelling to and attending frequent appointments as a “*bit of a hassle*” (YP4, aged 15).

Young people and health professionals identified needle phobia as a factor which may influence treatment decisions, this *“might put someone off [preventative bisphosphonate therapy]... having a needle injected and cannulation”* (YP17, aged 17, # aged 12).

Starting and managing new treatments could be anxiety-provoking for some parents: *“it’s a lot to take on when you start one form of medication, so the... thought of doing it at the same time as the steroids at the start, I think would have been a lot for us to worry about”* (P3, C aged 9, # aged 7).

The burden associated with preventative bisphosphonate therapy meant that some parents and health professionals were hesitant to have or recommend preventative therapy: *“if you can avoid it, avoid it and if everything else looks fine then avoid it”* (P15, C aged 14, # aged 11).

##### 3.2 “Beyond bisphosphonate”, fracture prevention is a “whole package of advice”

Health professionals and some parents believed “*there’s other things... that could be encouraged”* (P17, C aged 17 # aged 12) “*beyond bisphosphonate*” (HP3, consultant endocrinologist) for bone health protection and fracture prevention. They identified alternative non-invasive approaches which should be provided as a “*whole package of advice”* (HP2, specialist physiotherapist). Examples include monitoring and managing *“weight gain”* (HP3, consultant endocrinologist), “*having a specialist diet where it’s more high in vitamin D and like omegas... calcium... supplements*” (P18, C aged 11, # aged 9) and preserving mobility and muscle function: *“the biggest prevention would be movement… movement for as long as possible”* (HP26, consultant endocrinologist/bone metabolism).

Health professionals felt it was important for children/young people and parents to be aware of and understand fracture risks (for example, risky behaviours, safe transfers) to reduce the likelihood of fractures: *“weighing up some of the risks and not sort of taking part in risky behaviours... making sure they’re wearing their seat belts, not taking undue risks when transferring at that late ambulation stage... if we can actually prevent [fractures] from happening just through practical ways, that would be much better”* (HP2, specialist physiotherapist).

However, health professionals noted that there was also a lack of evidence or guidance on the effectiveness of these alternative preventative approaches: *“some families ask me whether taking calcium and vitamin D does help... the hard thing is again we don’t know... I can’t turn around and say I promise that if you keep the vitamin D above 75 your bones are going to definitely be better”* (HP26, consultant endocrinologist/bone metabolism).

New and innovative methods to monitor and manage bone health in DMD have been developed, such as vamorolone and “*better-quality imaging”* (HP26, consultant endocrinologists/bone metabolism), that may identify very early signs of bone fragility. Health professionals also questioned whether bisphosphonate treatments would be the best approach to bone protection and fracture prevention in the future: *“the newer bone sparing steroids such as vamorolone, using that more or going to develop more that kind of better anti-inflammatory for treatment would be one pathway that we need to lead... making sure that the muscle health is maintained better”* (HP27, neuromuscular clinician).

## Discussion

This is the first study to explore the perspectives and opinions of young people with DMD, parents and health professionals on the prospective acceptability of initiating preventative bisphosphonate therapy prior to fracture. Our findings revealed a continuum of opinions regarding provision of preventative IV bisphosphonate therapy. Parents’ opinions towards the use of preventative therapy were influenced by several factors. They include their access to and understanding of information about the therapy, the strength of the evidence base and the individual circumstances of children/young people (for example, ambulatory status). Overall, young people and parents were keen for preventative therapy if it could prevent and/or delay fractures. However, young people, parents and health professionals shared concerns about treatment burden or side-effects. Parents and health professionals also discussed fracture prevention treatment and bone health protection as being supported by the management of nutrition and the preservation of muscle function. Health professionals highlighted the importance of robust evidence to underpin treatment decisions in this population. There is a strong drive for research informed practice, as has been previously highlighted in other healthcare practices [34]. Health professionals therefore called for future research into the risks, benefits and effectiveness of preventative IV bisphosphonate therapy.

Preventative IV bisphosphonate therapy to improve bone health and/or prevent fractures may be acceptable to young people with DMD and parents. However, our study demonstrated that young people and parents valued participating in the choice whether or not to have a specific treatment. Choice for treatment was especially important where there was not strong underpinning evidence indicating a clear, favourable treatment approach. There is some evidence that IV bisphosphonate therapy can improve bone mineral density, alleviate back pain and prevent further progression of vertebral fracture in DMD when initiated following identification of fracture [15–17]. However, there is no available robust research evidence on the use and implications of providing IV bisphosphonate therapy preventatively in DMD, especially in relation to fracture outcome. There is also limited information on the sub-set of young people with DMD who are at the highest risk of imminent fracture. These young people may have the greatest need for the consideration of treatment prior to fracture.

Navigating shared decision-making in paediatric long-term health conditions is complex and involves consideration of the evidence alongside the views of all involved, taking into account concerns for treatment and individual circumstances [35–37]. Parents and young people with DMD in this study similarly discussed the balancing of decisions about preventative bisphosphonate therapy. Parents particularly wanted easy to understand and accessible information about preventative bisphosphonate therapy. Young people in our study did not discuss the use of information (such as research evidence) to inform their treatment decisions. Instead, they discussed the prospective acceptability of IV preventative bisphosphonate therapy if they thought it would benefit them, such as preventing fractures. This finding is similar to young people’s decision-making in other long-term health conditions (i.e., cancer) [37]. There is existing knowledge that young people with DMD and parents want to be better educated about and understand bone health [38]. However, they had concerns about the use of IV bisphosphonate therapy mainly due to side effects [39]. If IV bisphosphonates are to be provided preventatively as standard of care, this raises the need for the timely provision of accessible information (for example, in plain language) to families. One approach to help families make health decisions may be to provide them with patient decision aids which contain balanced information on the options and outcomes (risks, benefits) of treatments [40]. These aids could empower and assist children/young people and parents in making choices and decisions with health professionals about whether preventative therapy is appropriate for their current or anticipated circumstances. Parents and young people with DMD are commonly involved in making multiple choices and decisions along the trajectory of this long-term condition. Families weigh up many aspects of short and long-term monitoring and treatment, for example, linked to a child’s/young person’s muscles, weight, and respiratory function [41,42].

However, in our study, parents and health professionals specifically discussed difficulties with weighing up treatment decisions due to the current lack of evidence on the use of treatment prior to fracture. The additional difficulty in this setting is that IV bisphosphonates are associated with the risk of significant side-effects. Managing uncertainty when making treatment decisions is a common experience in paediatric long-term and complex health conditions [36,43,44]. Young people question their treatment decisions when experiencing uncertainty about treatment options or the risks and benefits of treatments [44]. Parents similarly struggle to make treatment decisions and face conflict about what is best for their child/young person when experiencing uncertainty [36]. Some parents trust healthcare professionals to make the right decision for their child/young person, particularly in instances of uncertain health outcomes [37,45].

Whilst this study was focussed on IV bisphosphonates, parents and health professionals discussed that oral administration of bisphosphonates for preventative therapy may be an acceptable alternative for families, compared to intravenous administration. Oral bisphosphonates are well tolerated by young people with DMD in a retrospective report [46], however, evidence is unclear on the long-term benefits of oral administration in improving bone mineral density [47–50]. There is even less information on the impact of oral bisphosphonates on fracture rate or on the use of oral bisphosphonates as a preventative treatment for osteoporosis in DMD [17,47,49,50]. The use of oral alendronate in young people with DMD following identification of fracture or in those with low and declining bone density led to stabilisation of vertebral fracture in the majority [49]. Twelve percent (5/43) developed new symptomatic vertebral fracture and 12% (5/43) with vertebral fracture had improvement in vertebral height. In a separate retrospective study, the use of oral risedronate in DMD prior to fracture was associated with a lower incidence of vertebral fractures compared to a historical control [51]. It is worth noting that there was no routine spine imaging for vertebral fracture in this study. Thus, future research into the effectiveness of oral bisphosphonates for preventing fractures and improving bone health in DMD is warranted.

A strength of this study is that collecting the opinions of young people, parents and health professionals enabled an in-depth exploration of multiple key stakeholders’ perspectives who are involved in clinical decision-making in this area. It was not a requirement for children/young people to have experienced a previous vertebral and/or long bone fracture. This was important for capturing a variety of perspectives given their experiences of having, or having not had a fracture and/or received bisphosphonate treatment, however the variability in the sample limited the conduction of any sub-group qualitative analysis.

The limitations of this study are that only a small number of young people shared their views about IV preventative bisphosphonate treatment and none were younger than 15 years. Some parents were present during young people’s interviews therefore young people may have provided responses which they thought aligned with their parent’s views. The recruitment approach and self-selecting sample may have led to the participation of young people, parents and professionals who held specific views about bone health and preventative treatment and management options. Whilst this study focussed on perspectives of preventative bone health therapy from within the UK, we believe the paper includes sufficient information and nuanced discussion for readers to evaluate the applicability or fit of the findings to other contexts [52] and offers transferable knowledge of use in subsequent studies [53].

## Conclusion

There was a continuum of opinions and perspectives regarding the prospective acceptability of preventative bisphosphonate therapy prior to fracture from young people with DMD, parents and health professionals. Parental choice for treatment is a personal decision which is influenced by their access to and understanding of information about the treatment, the strength of the evidence base and individual circumstances. The short- and long-term effects or harm on a child’s/young person’s health as a result of bisphosphonate therapy are a concern for parents and health professionals. Parents and health professionals called for additional and alternative non-invasive measures for fracture prevention and bone health protection. Future research is warranted to investigate the risks, benefits and effectiveness of using preventative bisphosphonate therapy in this population to inform clinical practice, as recommended by health professionals.

## Supporting information

S1 Table COREQ (COnsolidated criteria for REporting Qualitative research) Checklist. (DOCX)
