# Supplementary material for: The prospective acceptability of preventative IV bisphosphonate therapy prior to fracture: Perspectives of young people with Duchenne muscular dystrophy, parents and health professionals
Source: PLoS One. 2025 Jun 2;20(5):e0324124. doi: 10.1371/journal.pone.0324124 (PMC12129184; doi:10.1371/journal.pone.0324124)
Supplement: S1 Table — (DOCX) [file pone.0324124.s001.docx]

**Supplementary Table S1**

**COREQ (COnsolidated criteria for REporting Qualitative research) Checklist**

| **Topic** | **Item No.** | **Guide Questions/Description** | **Remarks** | **Page No.** |
| --- | --- | --- | --- | --- |
| **Domain 1: Research team and reflexivity** | | | | |
| *Personal characteristics* | | | | |
| Interviewer/ facilitator | 1 | Which author/s conducted the interview or focus group? | Two authors (DCM, LB) conducted the interviews and focus groups. | 7 |
| Credentials | 2 | What were the researcher’s credentials? E.g. PhD, MD | The researchers’ credentials are as follows:  DCM: MSc  NC: PhD  CLW: MD, PhD  SW: DMed(Sci)  LB: PhD | 7 |
| Occupation | 3 | What was their occupation at the time of the study? | The researchers’ occupations are as follows:  DCM: postdoctoral research assistant  NC: Consultant Clinical Scientist  CLW: Clinical Senior Lecturer / Consultant Paediatric Endocrinologist  SW: Clinical Senior Lecturer / Consultant Paediatric Endocrinologist  LB: Professor in Child Health Literacy | 7 |
| Gender | 4 | Was the researcher male or female? | The researchers’ genders are as follows:  DCM: female  NC: female  CLW: female  SW: male  LB: female | 7 |
| Experience and training | 5 | What experience or training did the researcher have? | DCM and LB are experienced researchers in qualitative studies. DCM, NC, CLW, SW and LB are experienced clinicians and/or researchers in paediatric long-term health conditions. | 7 |
| *Relationships with participants* | | | | |
| Relationship established | 6 | Was a relationship established prior to study commencement? | Relationships were established only for the purposes of the interviews/focus groups. The researchers spoke to parents via email and/or telephone/video call to arrange interviews and/or obtain further information, for example to check how much knowledge their child had about fractures. | 7 |
| Participant knowledge of the interviewer | 7 | What did the participants know about the researcher? E.g. personal goals, reasons for doing the research | The researchers (DCM, LB) explained the study aims and rationale to participants during recruitment and the consent process. | 7 |
| Interviewer characteristics | 8 | What characteristics were reported about the interviewer/ facilitator? E.g. bias, assumptions, reasons and interests in the research topic | The interviewers were not medical professionals who cared for young people with DMD. However DCM was aware of, and LB had worked in, the medical system. Both were aware of the literature on young people’s and parent’s experiences of DMD and bone health. | N/A |
| **Domain 2: study design** | | | | |
| *Theoretical framework* | | | | |
| Methodological orientation and theory | 9 | What methodological orientation was stated to underpin the study? E.g. grounded theory, discourse analysis etc | The research team conducted framework analysis and used inductive coding. | 8 |
| *Participant selection* | | | | |
| Sampling | 10 | How were participants selected? E.g. purposive, convenience, consecutive, snowball | We used a combination of convenience sampling followed by purposeful sampling to target children/young people, parents and health professionals from specific geographic areas and service provisions. Snowball sampling was encouraged by asking parents to share the study details with other potentially interested parents. | 6 |
| Method of approach | 11 | How were participants approached? E.g. face-to-face, telephone, mail, email | Study flyers for interviews were distributed on social media, through gatekeepers at relevant charities, parent networks and support groups. Study flyers for focus groups were distributed on social media and via Duchenne UK’s circulation list of clinicians through the DMD Care UK project. | 6-7 |
| Sample size | 12 | How many participants were in the study? | Fifty-one participants (four young people with DMD, twenty parents, twenty-seven health professionals) | 9 |
| Non-participation | 13 | How many people refused to participate or dropped out? Reasons? | Thirty-five parents expressed interest in taking part in an interview. Fifteen parents did not take part due to loss of contact (no reason for non-participation given). | N/A |
| *Setting* | | | | |
| Setting of data collection | 14 | Where was the data collected? E.g. home, clinic, workplace | Interview data was collected online (Teams) or at participant’s homes. Focus group data was collected online (Teams). | 7-8 |
| Presence of non-participants | 15 | Was anyone else present besides the participants and researchers? | Some young people chose to have parents present during their interview. | 8 |
| Description of sample | 16 | What are the important characteristics of the sample? E.g. demographic data, date | Important characteristics of the sample were children’s/young people’s age, approximate age at diagnosis, current corticosteroid regimen, approximate age started on corticosteroids, wheelchair use and experience of fracture. Other important characteristics were the parent’s relation to the child/young person and health professionals’ profession, the number of children/young people their centre managed and the corticosteroid regimens followed by children/young people in their centre. | 9 &  Table 1 |
| *Data collection* | | | | |
| Interview guide | 17 | Were questions, prompts, guides provided by the authors? Was it pilot tested? | The researchers provided children/young people and parents with example interview questions prior to the interview. Questions were not pilot tested but two parents from a UK DMD patient organisation guided the development of interview questions. | 7 |
| Repeat interviews | 18 | Were repeat interviews carried out? If yes, how many? | No repeat interviews were carried out. | N/A |
| Audio/visual recording | 19 | Did the research use audio and visual recording to collect the data? | Interviews and focus groups were audio-recorded. | 7-8 |
| Field notes | 20 | Were field notes made during and/or after the interview or focus group? | The two researchers (DCM, LB) made notes during focus groups, such as health professional demographic information (number of boys with DMD they managed at their clinical centre, the usual corticosteroid regimen prescribed at their clinical centre) | 7-8 |
| Duration | 21 | What was the duration of the interviews or focus groups? | Interviews lasted 12-20 minutes with young people and 20-45 minutes with parents. Focus groups lasted 66-83 minutes with health professionals. | 8 |
| Data saturation | 22 | Was data saturation discussed? | Data collection depended on data sufficiency, for example depending on the richness of the data collected including depth (i.e. to identify themes) and breadth (i.e. to identify discrepancies in accounts). | 7 |
| Transcripts returned | 23 | Were transcripts returned to participants for comment and/or correction? | Transcripts were not returned to participants. | N/A |
| **Domain 3: analysis and findings** | | | | |
| *Data analysis* | | | | |
| Number of data coders | 24 | How many data coders coded the data? | Two researchers (DCM, LB) coded the data. | 8 |
| Description of the coding tree | 25 | Did authors provide a description of the coding tree? | The two coders coded line by line to identify and create codes relevant to the research question. They discussed preliminary codes for the first five interviews and one focus group. They grouped these codes into cross-dataset categories and sub-categories. The researchers used this framework to index the remaining dataset. The framework was continually refined during discussions throughout the coding process. | 8 |
| Derivation of themes | 26 | Were themes identified in advance or derived from the data? | Themes were inductively derived from the data. | 8 |
| Software | 27 | What software, if applicable, was used to manage the data? | No specialised software was used to manage data. Data was managed using Microsoft Office (word, excel). | N/A |
| Participant checking | 28 | Did participants provide feedback on the findings? | Participants did not provide feedback on the findings. | N/A |
| *Reporting* | | | | |
| Quotations presented | 29 | Were participant quotations presented to illustrate the themes/findings? Was each quotation identified? E.g. participant number | Quotations were presented in a manner which protects participants’ confidentiality. | 10-19 |
| Data and findings consistent | 30 | Was there consistency between the data presented and the findings? | There was consistency between the data presented, which were supported by illustrative quotes, and the findings. | 10-19 |
| Clarity of major themes | 31 | Were major themes clearly presented in the results? | Major themes were clearly presented in the findings. | 10-19 |
| Clarity of minor themes | 32 | Is there a description of diverse cases or discussion of minor themes? | Themes on which there was a deviant opinion within groups (e.g. between parents) or between groups (e.g. between parents and health professionals) were discussed within the results and/or discussion. | 10-22 |
